# Surgical properties and survival of a pericardial window via left minithoracotomy for benign and malignant pericardial tamponade in cancer patients

**DOI:** 10.1186/1477-7819-10-123

**Published:** 2012-06-28

**Authors:** Sezai Celik, Muharrem Celik, Bulent Aydemir, Handan Tanrıkulu, Tamer Okay, Nurşen Tanrikulu

**Affiliations:** 1 Department of Thoracic Surgery, Siyami Ersek Thoracic and Cardiovascular Surgery Training and Research Hospital, Istanbul, Turkey; 2 Department of Anesthesiology and Reanimation, Siyami Ersek Thoracic and Cardiovascular Surgery Training and Research Hospital, Istanbul, Turkey; 3 Fatih Sultan Mehmet Mah. Balkan cad, Yesilvadi Evleri A3 Blok D: 36, Istanbul, Umraniye, 34771, Turkey

**Keywords:** Pericardial tamponade, Pericardial window, Cancer patients, Long-term survival

## Abstract

**Background:**

Surgical drainage is a rapid and effective treatment for pericardial tamponade in cancer patients. We aimed to investigate the effectiveness of pericardial window formation via mini-thoracotomy for treating pericardial tamponade in cancer patients, and to evaluate clinical factors affecting long-term survival.

**Methods:**

Records of 53 cancer patients with pericardial tamponade treated by pericardial window formation between 2002 and 2008 were examined. Five patients were excluded due to insufficient data. Kaplan-Meier and Cox regression analysis were used for analysis.

**Results:**

Forty-eight patients (64.7% male), with a mean age of 55.20 ± 12.97 years were included. Patients were followed up until the last control visit or death. There was no surgery-related mortality and the 30-day mortality rate was 8.33%; all died during postoperative hospitalization. Morbidity rate was 18.75%. Symptomatic recurrence rate was 2.08%. Cancer type and nature of the pericardial effusion were the major factors determining long-term survival (*P* <0.001 and *P* <0.004, respectively).

Overall median survival was 10.41 ± 1.79 months. One- and 2-year survival rates were 45 ± 7% and 18 ± 5%, respectively.

**Conclusion:**

Pericardial window creation via minithoracotomy was proven to be a safe and effective approach in surgical treatment of pericardial tamponade in cancer patients. Cancer type and nature of pericardial effusion were the main factors affecting long-term survival.

## Background

The prevalence of pericardial tamponade requiring surgical intervention is not uncommon in cancer patients and it is a life-threatening problem. It is possible to overcome this problem, prolong survival, and improve the quality of life of patients by effective management strategies. The essence of management is to achieve minimal morbidity and long-term survival without procedure-related mortality. The effectiveness of creating a pericardiopleural window via left minithoracotomy for the surgical treatment of pericardial tamponade in cancer patients, and the associated risk factors affecting long-term survival were investigated in this study.

## Methods

A total of 48 consecutive cancer patients with pericardial tamponade treated by creating a pericardial window via left mini-thoracotomy between February 2002 and January 2008 in our clinic were included in the study. The study was approved by the Local Ethics Committee. Patients, who underwent pericardiocentesis alone, those who had sclerotherapy along with pericardiocentesis, and those who were concurrently diagnosed with pericardial tamponade along with malignity, were excluded. During the study period, 22 patients with symptomatic pericardial effusion were treated by pericardiocentesis alone with no recurrence. These patients were directed to chemotherapy and mediastinal radiotherapy.

The mean age of the patients was 55.20 ± 12.97 years (range, 25–73 years), and 16 (33%) were female and 32 (64.7%) were male. All clinical information including the surgical procedure and complications, preoperative and postoperative echocardiography findings, cytopathological, cytogenetic, bacteriological, viral and immunological results, and patient outcome was retrospectively reviewed from patient files.

The diagnosis of pericardial tamponade was based on clinical features as well as the presence of at least one of the following criteria observed by standard M Mode, two-dimensional and Doppler echocardiography [1]: (1) right atrial collapse and right ventricular diastolic collapse; (2) an inspiratory decrease in peak mitral flow more than 30% (normal value, <10%), greater than 50% inspiratory increase in peak tricuspid inflow velocity (normal value, <25%); (3) dilated vena cava without any inspiratory collapse; (4) systolic anterior motion of the mitral valve; (5) swinging of the heart within the pericardium, ‘swinging heart’.

### Surgical technique

All patients were operated under general anesthesia with endotracheal intubation. The pericardium was exposed via a left minithoracotomy through the fourth intercostal space (a submammarian incision of 4 to 5 cm) using a pediatric chest wall retractor. After identification of the phrenic nerve, an anterolateral pericardial window of 4x4 or 5x5 cm was opened over the left ventricle, or if the tamponade was due to posterior fluid collection, a posterior pericardial window below the phrenic nerve was created. A chest drain (Nu. 28 F) was placed by a separate incision. The drain was removed after the daily drainage was reduced to below 100 mL.

### Follow-up

The follow-up of the patients were carried out by face-to-face interviews or over the phone until January 2008. Recurrence was defined as the subsequent occurrence of a symptomatic pericardial tamponade after pericardial window procedure. Survival was defined as the time from the window procedure until the last visit or time of death. Follow-up was performed in all patients, and the mean follow-up duration was 38.86 ± 9.63 months.

### Statistical method

Statistical analysis of the data was performed by using Number Cruncher Statistical System (NCSS) 2007&PASS 2008 statistical software (UT, USA). In addition to descriptive statistical methods, Kruskal Wallis test was used for the comparison of quantitative variables that were not normally distributed between the groups. Student *t* test was used for the comparison of normally distributed variables between two groups, and Mann Whitney *U* test was used for the comparison of non-normally distributed variables. The comparison of qualitative variables was performed using the chi-square test and Fisher’s exact chi-square test. The Kaplan-Meier method was used for survival analysis.

The prognostic value of the following variables were investigated: age, sex, primary malignancy, pericardial effusion-free interval, the amount and type of effusion drained during surgery, preoperative central venous pressure (CVP), hemoglobin and ejection fraction (EF) values, radiotherapy, distant metastasis, the presence and type of the accompanying pleural effusion, malignant or benign nature of the pericardial tamponade. Multivariate regression analysis of potential risk factors affecting long-term survival was performed by Cox regression analysis. A *P* value <0.05 was considered statistically significant.

## Results

All patients had a previous diagnosis of cancer. Pericardial tamponade occurred during ongoing chemotherapy in 32 patients, and after completion of chemotherapy, either while receiving mediastinal radiotherapy or within 2 months after this procedure, in 13 patients. Three patients were on simultaneous chemotherapy and radiotherapy when the tamponade occurred.

The etiology of pericardial effusions is presented in Table 1. Patient characteristics are presented in Table 2. In our series, 19 patients with symptomatic pericardial effusion underwent a single pericardiocentesis approximately 30 days before the surgery, and intrapericardial bleomycin was administered to four of them. However, these patients were re-admitted to our hospital with clinical signs of pericardial tamponade, and consequently treated with surgery. All patients in this series consented to undergo surgery. In general, the surgical procedure was well tolerated by all patients except one who developed hypotension during surgery. The operative time was 28.26 ± 3.76 min. The drainage volume during the operation and the postoperative period were 862.5 ± 390.37 mL and 450 ± 280 mL, respectively. The highest drainage volume was obtained from a lung cancer patient with malignant pericardial tamponade, and the lowest drainage volume was obtained from a cervix cancer patient with an idiopathic pericardial effusion with tamponade. While 37.5% of the pericardial effusion was hemorrhagic, 60.4% was serous and 2.1% was purulent. Pericardial effusion was benign in nature in 22 out of 48 patients (45.8%) and malignant in 26 (54.2%).

**Table 1:** The etiologies of pericardial effusions

**Diagnosis**	***n***
*Malignancy*	26
Lung cancer	14
Breast cancer	5
Hematological malignancy	4
Other	3
*Benign disorders*	22
Chronic non-specific pericarditis	15
Lung cancer	4
Breast cancer	3
Hematological malignancy	2
Nasopharyngeal cancer	1
Cervix cancer	1
Prostate cancer	1
Renal cell cancer	1
Testis cancer	1
Undifferentiated cancer	1
Tuberculosis	2
Mesothelioma	1
Hematological malignancy	1
Hypothyroidism	1
Renal cell cancer	1
Irradiation	2
Lung cancer	2
Bacterial pericarditis	1
Lung cancer	1
Viral pericarditis	1
Thymoma	1

**Table 2:** Demographic and preoperative data of the patients

	**Min-Max**	**Mean ± SD**
Male/female	32/16	64.7/33%
Age (years)	25-73	55.2 + 12.97
Pericardial effusion-free interval (months)	1-48	13.47 ± 10.34
Hb (g/dL)	9.6-16	11.65 ± 1.34
EF (%)	40-66	54.37 ± 5.89
Central venous pressure (cm H_2_0)	15-26	17 ± 7.6
	***n***	**%**
*Cancer type (n)*		
Breast	8	16.6
Hematologic	7	14.5
Lung	21	43.7
Other	12	25
*Appearance of effusion (n)*		
Hemorrhagic	18	37.5
Serous	29	60.4
Purulent	1	2.1
*Pericardial effusion type (n)*		
Malignant	26	54.2
Benign	22	45.8
*Accompanying pleural effusion (n)*	23	47.9
Malignant	12	52.2
Benign	11	47.8
Radiotherapy prior to or during tamponade	13	27
Chemotherapy prior to or during tamponade	48	100
Distant metastasis	14	35.9
Overt tamponade	48	100
Prior pericardiocentesis	19	40

SD, standard deviation.

No procedure-related mortality was observed in our study, and the symptomatic recurrence rate was found as 2.08% (1 patient). The 30-day mortality including in-hospital mortality was 8.33% (4 patients). The causes of 30-day mortality were ventricular fibrillation in one patient, pneumonia and sepsis due to prolonged intubation in one patient, pulmonary embolism in one patient, and low cardiac output in another. Morbidity rate was 18.75%. Information regarding morbidity is presented in Table 3.

**Table 3:** Operative and postoperative data of the patients

	
Operative time, min	28.26 ± 3.76
Drainage volume during surgery, mL	862.5 ± 390.37
Postoperative effusion drained, mL	450 ± 280
Duration of drainage, days	6.00 ± 1.46
Postoperative CVP (cmH_2_O)	9.5 ± 3.2
Length of hospital stay, days	9.5 ± 7.2
30-day mortality	4 (8.33%)
*Postoperative complications*	7 (14.5%)
Heart failure	1 (2.08%)
Arrhythmia	1 (2.08%)
Pulmonary embolus	1 (2.08%)
Pneumonia	1 (2.08%)
Prolonged ventilation	1 (2.08%)
Wound infection	2 (4.1%)
Follow-up, months	38.86 ± 9.63
Recurrence requiring intervention	1 (2.08%)

In order to improve hemodynamics, pericardiocentesis was performed in all patients just before the surgery, and pericardial window was opened subsequently. Symptomatic pericardial effusion recurred 1 month after the pericardial window procedure in one patient with epidermoid lung cancer (pericardial tamponade due to irradiation), who developed constrictive pericarditis with a small loculated effusion 1 month after the window procedure. Pericardiectomy was performed by median sternotomy in this patient who died due to low cardiac output and failure at postoperative day 20.

### Survival analysis

**Figure 1:**
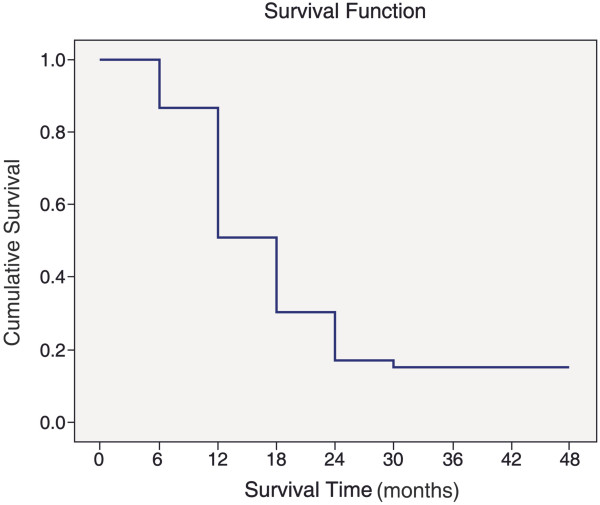
Survival for all patients.

Among 48 patients included in the study, four (8.3%) of them died during the hospitalization period and 36 (75%) died during follow-up. Eight patients (16.7%) were still alive at the end of the follow-up period. The mean survival time was 10.41 ± 1.79 months. One-year survival rate was 45 ± 7% and 2-year survival rate was 18 ± 5% (Figure 1).

**Figure 2:**
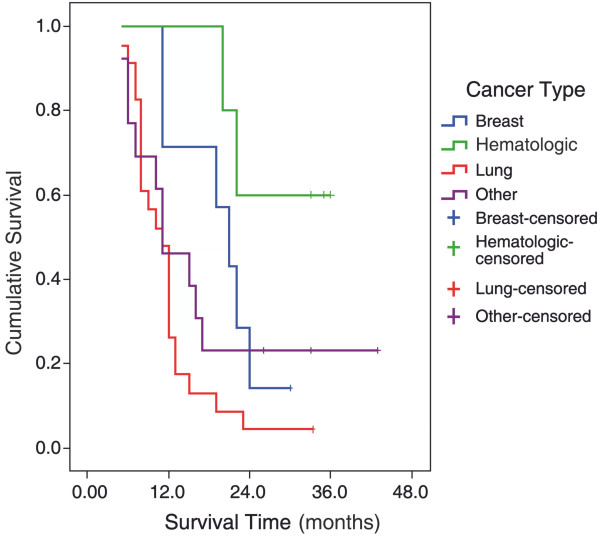
Survival for patients with cancer by tumor type.

**Figure 3:**
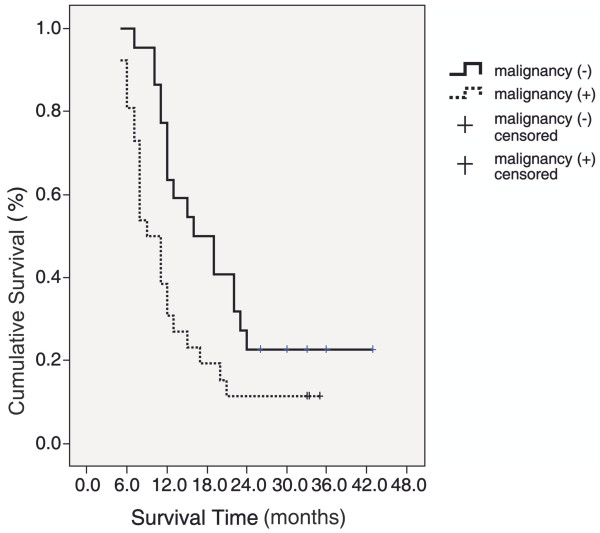
Survival for patients with malignant and benign tamponade.

The risk factors affecting survival are presented in Table 4. Overall survival did not differ significantly according to sex, the presence, appearance, and the type of the accompanying pleural effusion, radiotherapy, recurrence, and the presence of distant metastasis. However, a statistically significant difference was observed between the survival durations of patients with various cancer types (*P* <0.01). Comparison of paired groups revealed that survival time in the hematological malignancy group was significantly longer compared to that in lung cancer and other cancer groups (*P* = 0.001 and *P* = 0.019, respectively), and survival time in breast cancer group was significantly longer compared to that in lung cancer group (*P* = 0.014; Figure 2). Patients with malignant pericardial effusion had a significantly shorter survival time in compared to that of patients with benign pericardial effusion (*P* <0.01; Figure 3).

**Table 4:** Risk factors affecting survival

	**Survival (months)**		***P***
	**Mean ± SD**	**Median**	
*Gender*			
Female	17.43 ± 9.81	16	0.476
Male	15.07 ± 9.59	11	
*Cancer type*^b^			
Breast	19.71 ± 6.98	21	
Hematologic	29.20 ± 7.59	33	0.001^b^
Lung	11.79 ± 6.26	11	
Other	15.84 ± 11.57	11	
*Pleural effusion*			
Present	14.44 ± 8.38	11	0.420
Absent	17.16 ± 10.65	12	
*Macroscopic appearance of the effusion*			
Hemorrhagic	13.85 ± 10.12	11	0.136
Serous	17.24 ± 9.39	15	
*Nature of the pleural effusion*			
Malignant	14.91 ± 10.6	9.5	0.347
Benign	14.72 ± 6.19	12	
*Radiotherapy*			
Present	15.07 ± 8.20	12	0.701
Absent	17.16 ± 11.77	12.5	
*Distant metastasis*			
Present	14.73 ± 7.99	11.5	0.942
Absent	14.80 ± 8.41	12	
*Nature of the pericardial effusion*			
Malignant	11.89 ± 8.78	9.5	0.004*
Benign	18.36 ± 9.58	16.5	

SD, standard deviation.

Mann Whitney *U* test.

^a^Kruskal Wallis test.

^b^*P* <0.01.

The results of correlation analysis between quantitative parameters and survival are presented in Table 5.

**Table 5:** Results of correlation analysis between quantitative parameters and survival

	**Survival (months)**	
	**r**	***P***
Age	−0.401	0.005^a^
Amount of effusion removed during surgery (mL)	−0.220	0.134
Preoperative Hb (g/dL)	0.463	0.001^a^
Preoperative EF (%)	0.193	0.189
Pericardial effusion-free interval (months)	0.292	0.044^b^
Preoperative CVP (cmH20)	0.174	0.168

CVP, central venous pressure; EF, ejection fraction; Hb, hemoglobin.

R, Spearman’s rho coefficient.

^a^*P* <0.01.

^b^*P* <0.05.

A statistically significant negative correlation was found between age and survival time (r = −0.401, *P* = 0.005). Survival time decreased with increasing age. Hemoglobin levels and survival time was positively correlated (r = 0.463, *P* = 0.001). Furthermore, there was a statistically significant positive correlation between the pericardial effusion-free interval and survival time (r = 0.292, *P* = 0.044). No significant correlation was noted between survival time and the amount of effusion removed, preoperative CVP, and EF (*P* >0.05 for all parameters).

The variables, which were found to be significant or close to significance by univariate analysis, were evaluated by Cox regression analysis. Age above 55 years and lung cancer were found as significant factors affecting survival. Lung cancer increased the risk of mortality by 1.997-fold (95% CI: 1.004-3.973). Although age was a significant risk factor, the odds ratio for age was only 0.351 (95% CI: 0.175-0.704) (Table 6).

**Table 6:** Results of univariate and multivarite Cox Regression analysis for potential risk factors affecting survival

	**Univariate**				**Multivariate (Cox Regression)**			
	***P***	**Odds**	**95% CI**		***P***	**Odds**	**95% CI**	
			**Lower**	**Upper**			**Lower**	**Upper**
Age >55 years	0.002^a^	0.607	0.502	0.885	0.003^b^	0.351	0.175	0.704
EF <50	0.210	3.769	0.420	33.80	0.221	1.536	0.773	3.055
Lung cancer	0.028^b^	8.556	1.161	77.13	0.045^b^	1.997	1.004	3.973

^a^*P* < 0.01.

^b^*P* <0.05.

EF, ejection fraction.

Assessment of survival rates according to the nature of pericardial effusion by Log Rank test revealed a significant difference (*P* = 0.019; *P* <0.05). The survival time was shorter in patients with malignant effusions (Figure 3).

## Discussion

Pericardial effusion is less common than pleural effusion in cancer patients; however, it often has an acute onset, and may be a direct cause of death if it leads to tamponade, and is not effectively treated. The ideal management of this life-threatening condition involves urgent decompression of the pericardial cavity, obtaining sufficient amount of fluid and tissue sample for the identification of the exact cause of tamponade, prevention of recurrence, and targeting a long survival period with the accomplishment of all of these goals with the lowest possible morbidity and mortality. Principally, pericardiocentesis procedure does not fulfill these criteria due to high recurrence rates [2-4]. Recent studies have suggested surgical pericardial window procedure instead of pericardiocentesis in patients with pulmonary adenocarcinoma who achieve a longer life expectancy by chemotherapy [5]. [Bitran et al.] reported that creation of a pericardial window improved survival time in breast cancer patients who developed pericardial tamponade. However, it might still be necessary to perform pericardiocentesis prior to pericardial window procedure to prevent sudden low cardiac output. It has been reported by [Olsen et al.] that creating a pericardial window through left minithoracotomy is a rapid, simple, and inexpensive technique, which we have also been routinely performing for a long period of time, especially in cases of pericardial tamponade. [Olsen et al.] created a pericardial window in 28 patients with large malignant pericardial effusions using this method and reported that the postoperative mortality was 21%. There were no procedure-related intra- or postoperative death, and symptomatic recurrence was noted in only one patient. We particularly included cancer patients with pericardial tamponade in our study, as there is currently no sufficient information or consensus regarding the management of this condition in cancer patients. [Gregory et al.] treated pericardial tamponade by creating a window through anterolateral thoracotomy in 49 cancer patients, and reported the in-hospital mortality as 8% and the rate of recurrence as 6%. We did not observe any procedure-related mortality in our series and the recurrence rate was 2.08%. The 30-day mortality including in-hospital mortality was 8.33% (4 patients). The patient who died due to pneumonia and sepsis secondary to prolonged intubation had both chronic obstructive pulmonary disease and a body mass index (BMI) >28. Another patient who died of pulmonary embolism in this group had a previous history of pulmonary embolism. Death occurred due to ventricular fibrillation in one patient with a previous history of ischemic heart disease, and low cardiac output in another patient with chronic ischemic heart disease. No late pericardial constriction was noted in patients with long survival rates.

The major reason for preferring left minithoracotomy for creating a pericardial window in our clinic for years is that this group of patients need prompt and definitive diagnosis and treatment. This technique is advantageous as it does not require single-lung ventilation, it can be applied in only 25 to 30 min, it provides the opportunity for opening a larger window, thus allows obtaining a larger tissue sample with a greater chance of histopathological diagnosis, and it offers the opportunity for pleural biopsy. Moreover, mini-submammary incision is much more cosmetic compared to subxiphoid lengthwise incision. The most important disadvantage of the technique is the risk of sudden hypotension during induction of general anesthesia, but this risk can be overcome by a pericardiocentesis performed right before the procedure. For this reason, echo-guided pericardiocentesis was performed in all patients before the pericardial window procedure in our study, and no intraoperative problems were encountered in these patients. Postoperative pain can easily be controlled via a patient-controlled anesthesia pump. According to our experience, pericardial window procedure is associated with a specific disadvantage, which is the difficulty of performing left minithoracotomy in breast cancer patients with previous left mastectomy and subsequent radiotherapy. The presence of excessive adhesions involving the pericardium, mediastinal tissues and chest wall is highly likely in these cases, and this might increase the odds of myocardial rupture during thoracotomy and exploration. Moreover, it might also be difficult to perform minithoracotomy in obese individuals and women with large breasts. Other methods should be considered in these cases.

Video-assisted thoracic surgery (VATS) procedure is nearly impossible, especially in cases where single-lung ventilation cannot be performed due to hemodynamic instability. Actually, the number of studies on VATS pericardial window in hemodynamically unstable cancer patients with pericardial tamponade remains limited. This indicates that the effective use of VATS in this patient group continue to be a problem. In their study, [Georghiou et al.] created a pericardial window via VATS in 18 patients and stated that VATS had the advantages of both subxiphoid pericardiotomy and pericardiectomy. In their series, however, there was no hemodynamically unstable patient with cardiac tamponade. [Fibla et al.] reported that eight of twelve patients with cancer who had pericardial tamponade were treated effectively with VATS. As we apply VATS in selected patients, these patients have not been included in the present study.

Although decompression of the pericardial cavity by subxiphoid approach under local anesthesia is an easier technique to perform, it is essentially equivalent to tube thoracostomy, and is associated with high recurrence rates. Therefore, creating a pericardial window by subxiphoid method should not be preferred in patients with a high probability of long-term survival. In the study by [O’Brien et al.], in which subxiphoid approach and VATS were compared, patients with hemodynamic instability did not constitute the majority. They reported the recurrence rate of pericardial effusion to be 8% after VATS. In a prospective study [12] including 30 patients, of which 15 (10 with tamponade) underwent VATS and 15 (seven with tamponade) underwent surgical procedure (transthoracic or subxiphoid), no significant difference was found in terms of intra- or postoperative complications, mortality, duration of drainage, duration of hospitalization, and recurrence; however, operative time was reported to be significantly longer in those undergoing VATS. [Becit et al.] reported a recurrence rate of 10% within 1 month of subxiphoid surgical pericardiostomy in 368 patients with pericardial effusions (most of them had pericarditis associated with tuberculosis and uremia) and they created a pericardial window by left anterior thoracotomy in these patients with no subsequent recurrence. [Mueller et al.] reported an overall success rate of 82% for subxiphoid pericardial window procedure in their series of 67 patients (26 cancer-related; 14 malignant pericardial effusions). In a prospective study from Duke University, surgical subxiphoid pericardiotomy was done under local anesthesia in 77% of 57 patients with various diseases, with general anesthesia required in the others. Effusion recurred in eight patients in 2 months and in nine (16%) in the first year.

We also found that the presence of malignant pericardial tamponade was a negative risk factor for long-term survival. Contradictory findings have been reported in previous studies using various surgical techniques, some supporting [14,15] and some not supporting [16,17] our results. [Wang et al.] reported that there was no significant difference between survival rates of malignant (*n* = 60) and benign (*n* = 20) pericardial effusion cases. We found a significant difference between these groups in our study. It should also be noted that the overall median survival found in our study was longer compared to previous reports. [Olsen et al.] reported in patients with malignant effusion treated by minithoracotomy pericardial window that the 6-month survival rate was 46%, the 1-year survival rate was 26%, and the 2-year survival rate was 15%. These results are similar to the median survival rates noted in our series. However, the factors affecting survival were not analyzed in their study.

On the other hand, the use of minimally invasive treatments has been previously studied in this group of patients; however, these treatments have not been currently implemented in routine clinical practice due to various reasons. Drainage with percutaneous catheter in patients with cardiac tamponade can be applied in the intensive care units and is a less invasive method. Nonetheless, the associated complication and recurrence rates are high. In their series of 42 patients, [Kopecky et al.] reported the complication rate to be 2.4% and the recurrence rate to be 24%. In the study by [Chow et al.], pericardial window formation via percutaneous balloon catheter was defined; however, sufficient experience has not yet been gained for the use of this method.

The mean survival time in hematological malignancies was found as 29.20 ± 7.59 (33) months in our study. Similar results were also reported in studies using subxiphoid pericardial window technique [21]. Certainly, the long survival times in our patients can primarily be attributed to the advances in systemic treatment. The high number of cases with solid organ cancers in our study population can also explain our findings.

Currently, no standardization exists among chest surgeons as to which procedure should be used to treat pericardial tamponade in cancer patients. Based on our findings, we suggest that creating a pericardial window by minithoracotomy should be preferred as a definitive surgical procedure with the lowest recurrence rates, especially in patients with long-term survival expectancy.

Our study has several limitations including its retrospective nature, the relatively small sample size, and heterogeneity of the study population, which limits the generalization of our findings to the general population.

## Conclusions

In conclusion, creation of a pleuropericardial window by left minithoracotomy provides a prompt, safe, and effective diagnostic and treatment approach, especially in selected cases of both malignant and benign pericardial tamponade occurring in cancer patients. Moreover, the likelihood of recurrence of pericardial effusion is rather low with this technique. Type of the underlying cancer and nature of the pericardial effusion were noted as the main factors affecting long-term survival in our patients.

## Abbreviations

BMI: Body mass index; CVP: Central venous pressure; EF: Ejection fraction; NCSS: Number cruncher statistical system; VATS: Video-assisted thoracic surgery.

## Competing interest

The authors declare that they have no conflict of interest.

## Authors’ contribution

SC: design, acquisition of data, analysis and interpretation of data, have given final approval of the version to be published. MC: have been involved in drafting the manuscript or revising it critically for important intellectual content, analysis of data, participated in the sequence alignment. BA: conceived of the study, and participated in its design and coordination and helped to draft the manuscript. HT: participated in the design of the study and performed the statistical analysis. TO: have made substantial contributions to conception and design, or acquisition of data, or analysis and interpretation of data. NT: participated in the design of the study and performed the statistical analysis. Involved in drafting the manuscript. All authors read and approved the final manuscript.
